# Supplementary material for: Increased Frequency of Circulating Activated FOXP3+ Regulatory T Cell Subset in Patients with Chronic Lymphocytic Leukemia Is Associated with the Estimate of the Size of the Tumor Mass, STAT5 Signaling and Disease Course during Follow-Up of Patients on Therapy
Source: Cancers (Basel). 2024 Sep 22;16(18):3228. doi: 10.3390/cancers16183228 (PMC11430700; doi:10.3390/cancers16183228)
Supplement: Supplementary file 1 [file cancers-16-03228-s001.zip › Suppl. Table S1.pdf]

Supplementary Table S1: Characteristics of CLL patients not included in the follow up study

	Gender	Age	Disease stage	Previous therapy
Patient 1	male	62	Binet A	0
Patient 2	male	74	Binet A	0
Patient 3	male	61	Binet A	0
Patient 4	male	64	Binet A	0
Patient 5	male	70	Binet A	0
Patient 6	male	58	Binet A	0
Patient 7	female	69	Binet B	0
Patient 8	male	69	Binet A	0
Patient 9	male	72	Binet A	0
Patient 10	male	83	Binet A	0
Patient 11	male	80	Binet A	0
Patient 12	male	80	Binet A	0
Patient 13	male	77	Binet B	0
Patient 14*	female	78	Binet A	BTKi
Patient 15	male	71	Binet A	0
Patient 16*	male	56	Binet A	CIT
Patient 17*	male	81	Binet A	BTKi
Patient 18	male	84	Binet A	0
Patient 19	female	69	Binet A	0

Abbrevations: CIT, chemo-immunotherapy; BTKi, Bruton Tyrosine Kinase inhibitor, * patients treated before enrollment
