# Supplementary material for: Increased Frequency of Circulating Activated FOXP3+ Regulatory T Cell Subset in Patients with Chronic Lymphocytic Leukemia Is Associated with the Estimate of the Size of the Tumor Mass, STAT5 Signaling and Disease Course during Follow-Up of Patients on Therapy
Source: Cancers (Basel). 2024 Sep 22;16(18):3228. doi: 10.3390/cancers16183228 (PMC11430700; doi:10.3390/cancers16183228)
Supplement: Supplementary file 1 [file cancers-16-03228-s001.zip › Suppl. Table S2.pdf]

Supplementary Table S2: Characteristics of controls included in the study

	Gender	Age	Al., A.I., A.D.*	Immunosuppressive therapy
Control 1	female	54	No	0
Control 2	male	76	No	0
Control 3	female	58	No	0
Control 4	female	73	No	0
Control 5	female	56	No	0
Control 6	female	55	No	0
Control 7	female	54	No	0
Control 8	female	83	No	0
Control 9	female	54	No	0
Control 10	female	58	No	0
Control 11	female	61	No	0
Control 12	female	57	No	0
Control 13	female	54	No	0
Control 14	female	63	No	0
Control 15	male	52	No	0
Control 16	male	53	No	0
Control 17	female	63	No	0
Control 18	female	51	No	0
Control 19	female	54	No	0
Control 20	male	78	No	0

Abbreviations: Al., Allergy; A.I., Acute infection; A.D., Autoimmune Disease; *
